# Bovine and Human Tuberculosis

**Published:** 1901-09

**Authors:** 


### EDITORIAL NOTES AND COMMENTS.

---

The Business office of THE JOURNAL-RECORD is No. 64 Marietta Street.

The Editorial office is 318-319-320 The Prudential.

Address all Business communications to Dr. M. B. Hutchins, Mgr.

Make remittances payable to THE ATLANTA JOURNAL-RECORD OF MEDICINE.

On matters pertaining to the Editorial and Original Communications address Dr. Bernard Wolff, Atlanta.

Reprints of original articles will be furnished at cost price. Requests for the same should always be made *on the manuscript*.

We will present, post-paid, on request, to each contributor of an original article, twenty (20) marked copies of THE JOURNAL-RECORD containing such article.

---

### BOVINE AND HUMAN TUBERCULOSIS.

KOCH'S denial of the identity of human and bovine tuberculosis which has taken up so much space in the medical and secular press, is one of the gifts of science of equivocal value. The world has been roused to interest in Koch's discoveries before, and there are not lacking those incredulous spirits who will take the news with a dash of salt in it. It may or may not be correct. The world and human kind have not reached the point where voluntary sacrifice of human life is feasible, and for this reason the problem will remain on the knees of the gods until such a time as some one with more philanthropy than sense shall offer his person for making the experiment of inoculation with bovine tubercle.

If by peradventure, Koch's statement be true, error is more to be desired than truth. It does not take much thought to discern the subversive effect that this change of conception would produce if carried out practically upon important departments of sanitary science. When so much has been done for the protection of the public against the hitherto accepted danger of tuberculous meat, and so much money has been spent and is being spent in this alleged illusory precaution, would it not be better to persist in error than to desist from truth? Certainly tuberculous meat is not desirable as an article of diet any more than is slink veal or measly pork, and this argument should suffice to prevent any relaxation of protection. Here are some of Koch's conclusions.

I have reached the conclusion that mankind's fear of contact with tuberculosis-infected flesh and fluid is unnecessary and unfounded.

I arrived at the discovery through what I consider practically indisputable tests. These experiments lead me to believe that human tuberculosis and bovine tuberculosis are two entirely distinct species.

I have found the human tuberculon incapable of inoculation into the animal system. Proceeding from that premise, I am prepared to show that humanity's far-reaching precautions against infected cattle may once for all be abandoned.

So it is that one by one the "truths" of science are found to be only relatively true and the foundation of many of our beliefs are nibbled and gnawed at until they fall. What was an accepted fact to-day, to-morrow is an exploded theory. It gives a feeling of uncompleteness and insecurity in keeping with modern pessimism. "*Tout lasse, tout passe.*"

